# Functionally distinct disease-associated fibroblast subsets in rheumatoid arthritis

**DOI:** 10.1038/s41467-018-02892-y

**Published:** 2018-02-23

**Authors:** Fumitaka Mizoguchi, Kamil Slowikowski, Kevin Wei, Jennifer L. Marshall, Deepak A. Rao, Sook Kyung Chang, Hung N. Nguyen, Erika H. Noss, Jason D. Turner, Brandon E. Earp, Philip E. Blazar, John Wright, Barry P. Simmons, Laura T. Donlin, George D. Kalliolias, Susan M. Goodman, Vivian P. Bykerk, Lionel B. Ivashkiv, James A. Lederer, Nir Hacohen, Peter A. Nigrovic, Andrew Filer, Christopher D. Buckley, Soumya Raychaudhuri, Michael B. Brenner

**Affiliations:** 1 Division of Rheumatology, Immunology, and Allergy, Brigham and Women’s Hospital, Harvard Medical School, Boston, MA 02115 USA; 2 Division of Genetics, Brigham and Women’s Hospital, Harvard Medical School, Boston, MA 02446 USA; 3 Broad Institute of MIT and Harvard, Cambridge, MA 02142 USA; 4 Bioinformatics and Integrative Genomics, Harvard University, Cambridge, MA 02138 USA; 5 Department of Biomedical Informatics, Harvard Medical School, Boston, MA 02115 USA; 6 Rheumatology Research Group, Institute of Inflammation and Ageing (IIA), University of Birmingham, Queen Elizabeth Hospital, Birmingham, B15 2WB UK; 7 Department of Orthopedic Surgery, Brigham and Women’s Hospital, Boston, MA 02115 USA; 8 Arthritis and Tissue Degeneration Program and the David Z. Rosensweig Genomics Research Center, Hospital for Special Surgery, New York, NY 10021 USA; 9 Weill Cornell Graduate School of Medical Sciences, New York, NY 10021 USA; 10 Department of Surgery, Brigham and Women’s Hospital and Harvard Medical School, Boston, MA 02115 USA; 11 Center for Immunology and Inflammatory Diseases, Massachusetts General Hospital, Charlestown, MA 02114 USA; 12 Department of Medicine, Harvard Medical School, Boston, MA 02115 USA; 13 Division of Immunology, Department of Medicine, Boston Children’s Hospital, Harvard Medical School, Boston, MA 02115 USA; 14 Arthritis Research UK Centre for Genetics and Genomics, Manchester Academic Health Science Centre, University of Manchester, Manchester, M13 9PT UK; 15 Present Address: Department of Rheumatology, Graduate School of Medical and Dental Sciences, Tokyo Medical and Dental University (TMDU), Tokyo, 113-8519 Japan; 16 Present Address: JW Creagene Corporation, Seongnam-Si, 13202 South Korea; 17 Present Address: Division of Rheumatology, University of Washington, Seattle, WA 98109 USA

## Abstract

Fibroblasts regulate tissue homeostasis, coordinate inflammatory responses, and mediate tissue damage. In rheumatoid arthritis (RA), synovial fibroblasts maintain chronic inflammation which leads to joint destruction. Little is known about fibroblast heterogeneity or if aberrations in fibroblast subsets relate to pathology. Here, we show functional and transcriptional differences between fibroblast subsets from human synovial tissues using bulk transcriptomics of targeted subpopulations and single-cell transcriptomics. We identify seven fibroblast subsets with distinct surface protein phenotypes, and collapse them into three subsets by integrating transcriptomic data. One fibroblast subset, characterized by the expression of proteins podoplanin, THY1 membrane glycoprotein and cadherin-11, but lacking CD34, is threefold expanded in patients with RA relative to patients with osteoarthritis. These fibroblasts localize to the perivascular zone in inflamed synovium, secrete proinflammatory cytokines, are proliferative, and have an in vitro phenotype characteristic of invasive cells. Our strategy may be used as a template to identify pathogenic stromal cellular subsets in other complex diseases.

## Abstract (web summary)

Synovial fibroblasts are thought to be central mediators of joint destruction in rheumatoid arthritis (RA). Here the authors use single-cell transcriptomics and flow cytometry to identify synovial fibroblast subsets that are expanded and display distinct tissue distribution and function in patients with RA.

## Introduction

Fibroblasts are important mediators of end-organ pathology and inflammation in chronic inflammatory and fibrotic diseases. Although these cells mediate normal matrix deposition and inflammation in wound healing, chronically activated fibroblasts can differentiate into myofibroblasts that produce collagen and are required for fibrosis in lung, liver, gut, skin, and other tissues^1^. Conversely, chronically activated fibroblasts are responsible for excessive matrix degradation that destroys cartilage and causes permanent joint damage in rheumatoid arthritis (RA)^2–4^. Moreover, studies have emphasized the role of fibroblasts as stromal cells that regulate immune responses in lymph nodes and tumor stroma^5,6^. Unlike hematopoietic cell types that are comprised of a variety of functionally distinct cellular types and subsets, fibroblasts are generally considered to have little heterogeneity; functionally distinct subpopulations have yet to be clearly defined.

Advances in high-throughput technologies have enabled investigators to query complex human diseases in new ways. For example, global transcriptomic analysis has revealed distinct activation states and cellular subsets of immune cells^7^. These approaches offer an opportunity to examine how stromal cells mediate various types of local tissue pathology. Transcriptomics of small numbers of cells, and even single cells, from human pathology samples can advance the understanding of tissue dynamics in disease. For example, single-cell RNA-sequencing (RNA-seq) identified heterogeneity of tumor cells and a mechanism for drug resistance in cancer^8,9^.

RA is a complex autoimmune disease affecting up to 1% of the world’s population^10^. In RA, the synovium changes dramatically as the thin membrane encapsulating the joint becomes an inflamed, hyperplastic, and invasive tissue mass that causes joint destruction^4^. Synovial fibroblasts secrete inflammatory cytokines and chemokines, invade and degrade cartilage, and stimulate osteoclasts that cause bone erosion^2,4^.

Here we show that these different functions might be carried out by distinct cellular subsets of fibroblasts, analogous to functionally distinct subsets of leukocytes. We propose that altered proportions of fibroblast subsets might underlie pathological changes in joint tissues^11^. We use flow cytometry to profile the abundance of fibroblast subsets in fresh human tissues from arthroplasty surgeries of patients with late-stage or early-stage disease. We use transcriptomics to define gene signatures that distinguish subpopulations of fibroblasts and predict their normal and pathological activity. We use histological images with immunofluorescence staining to visualize the microanatomy of the synovial tissue and localize fibroblast subsets in the lining layer, sublining layer, and perivascular regions. Finally, we perform functional experiments to identify which fibroblast subsets are more likely to carry out different molecular functions such as osteoclastogenesis and monocyte recruitment.

## Results

### Fibroblasts in synovial tissue have distinct surface markers

To examine the heterogeneity of fibroblasts in joint tissue, synovial cells were isolated from tissues collected from joint replacement surgery of patients with RA or osteoarthritis (OA), and from synovectomies of patients with RA. Osteoarthritic changes can be present in RA patients at the late stage of disease when it is time for joint replacement surgery. Taking this into consideration, we reasoned that comparison with synovial tissue from OA patients would reveal autoimmune changes specific to RA that were different from those found in comparator OA samples.

**Fig. 1:**
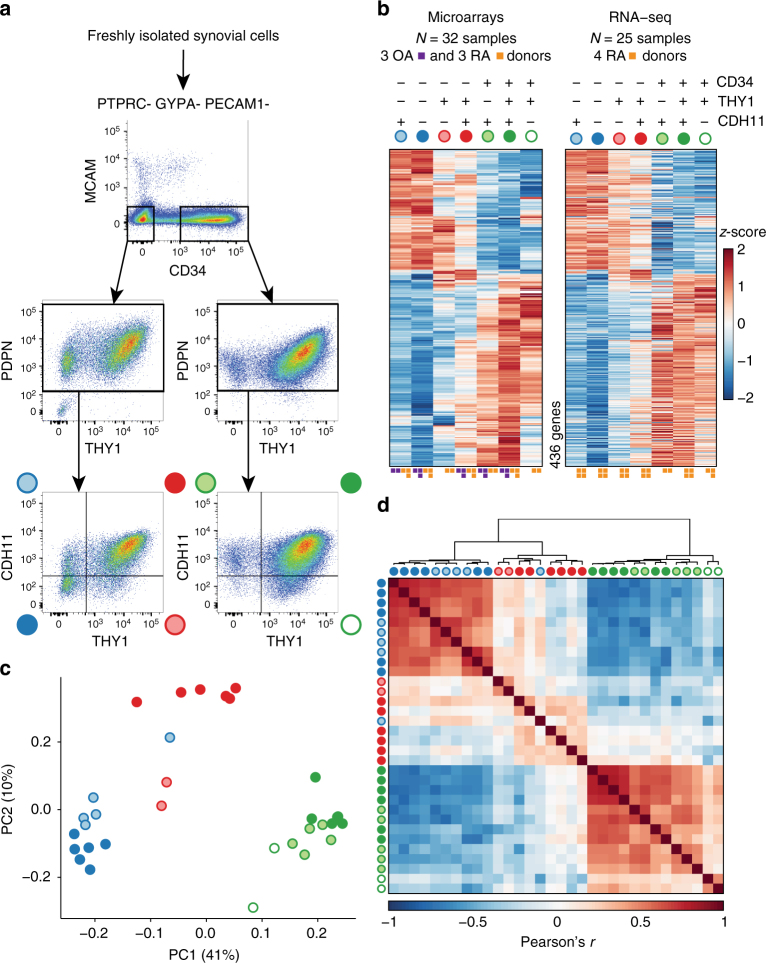
Distinct protein and mRNA expression between fibroblast subsets. **a** Gating strategy for synovial fibroblasts with heterogeneous expression of surface proteins. **b** Analysis of variance (ANOVA) reveals 436 genes with significant (1% FDR) variation across seven gated populations that are measured and statistically significant in both microarray and RNA-seq datasets. Each column in the heatmap corresponds to the average of multiple samples of a cell sorting gate. Each square beneath a column represents a donor from which this sample was taken. **c** Principal component analysis (PCA) with 2,986 genes (1% FDR, ANOVA) in microarray data separates the 32 microarray samples into three subsets: CD34^–^THY1^–^, CD34^–^THY1^+^, and CD34^+^. **d** Pairwise Pearson's correlation of microarrays also suggests three major subsets of fibroblasts

We first examined freshly isolated synovial fibroblasts for protein expression of a variety of surface markers that have been reported to be expressed on fibroblasts^12–17^. After screening many surface proteins, we chose podoplanin (PDPN) and cadherin-11 (CDH11) because of their characteristic expression on fibroblasts and THY1 (also known as CD90) and CD34 for their ability to distinguish fibroblast subpopulations (Fig. 1a). We isolated synovial fibroblasts by excluding other cells^17^. We excluded hematopoietic lineage cells positive for protein tyrosine phosphatase receptor type C (PTPRC, also known as CD45). We excluded red blood cells positive for glycophorin A (GYPA, also known as CD235a). We excluded endothelial cells positive for platelet and endothelial cell adhesion molecule 1 (PECAM1, also known as CD31). Finally, we excluded pericytes positive for cell surface glycoprotein MUC18 (MCAM, also known as CD146). The remaining stromal cells exhibited high protein PDPN expression, consistent with fibroblasts within the synovium (Fig. 1a). Two major fibroblast populations were identified based on surface protein expression of CD34. In 42 donors (26 OA and 16 RA), we observed medians of 34.7% CD34^+^ and 54.7% CD34^–^ cells (Fig. 1a, Supplementary Table 1). CD34^–^ and CD34^+^ fibroblasts could further be divided into four and three populations, respectively, based on THY1 and CDH11 expression.

### Fibroblast subpopulations have distinct mRNA signatures

To test whether these subsets represent distinct fibroblast populations, we applied two complementary strategies to investigate synovial fibroblast populations obtained after tissue disaggregation. The first strategy utilizes fluorescence sorting using a set of candidate protein markers followed by transcriptomic profiling of gated populations. The second strategy uses unbiased single-cell transcriptomics without gating.

First, we assayed each of the seven gated populations from three OA and three RA donors with the Affymetrix HuGene 2.0 ST microarray, using Robust Multichip Average to normalize 53,617 probesets and 20,452 genes. As expected, we observed that all of the samples expressed genes typically expressed in fibroblasts and lacked expression of other lineage-specific genes (Supplementary Fig. 1A). However, after controlling for variation between donor, we observed 2,986 genes with significant variation across the seven distinct populations gated based on surface protein marker expression (false discovery rate (FDR) <1%, analysis of variance (ANOVA)), suggesting notable transcriptional differences across these putative subsets. To validate these microarray findings, we applied RNA-seq to the same seven subsets from four independent RA donors. We prepared libraries with Smart-Seq2, sequenced to an average depth of 5.6M fragments per sample, and quantified expression for 19,532 genes. These samples were also enriched with fibroblast lineage genes (Supplementary Fig. 1B). Those genes that were significantly differentially expressed in the microarray experiment and that overlapped with the RNA-seq experiment (*n* = 2,659 genes) had similar expression profiles across the seven subsets (Supplementary Fig. 2). In total, 436 genes were measured on both platforms and had significant variation across the seven putative subpopulations in both experiments (1% FDR, ANOVA) (Fig. 1b). The concordance of these results suggests that the gene expression differences reflect biological variation rather than technical or stochastic artifacts. We reasoned that these expression profiles could serve as proxies for molecular functions to define putative cellular subsets with distinct biological roles.

Principal component analysis (PCA) of the microarray data revealed that seven phenotypic populations fall into three distinct major subsets: CD34^–^THY1^–^, CD34^–^THY1^+^, and CD34^+^. Principal component 1 clearly separates CD34^–^ and CD34^+^ samples and PC2 separates the CD34^–^ samples that are THY1^+^ and CDH11^+^ (Fig. 1c). We observed the same pattern in the RNA-seq data (Supplementary Fig. 3). The three subsets were also clearly apparent by hierarchical clustering on pairwise Pearson's correlations of gene expression profiles (Fig. 1d). CD34^–^ samples were positively correlated with each other and CD34^+^ samples were also positively correlated with each other, but CD34^–^ and CD34^+^ samples were negatively correlated. CD34^–^THY1^+^ sample correlations with the other samples were less consistent, indicating that this subset may be more heterogeneous than the CD34^–^ subset or the CD34^+^ subset (Fig. 1d). We decided to group CD34^–^THY1^–^CDH11^–^ and CD34^–^THY1^–^CDH11^+^ samples together because they had similar gene expression profiles overall.

### Single-cell RNA-seq identifies three major fibroblast subsets

**Fig. 2:**
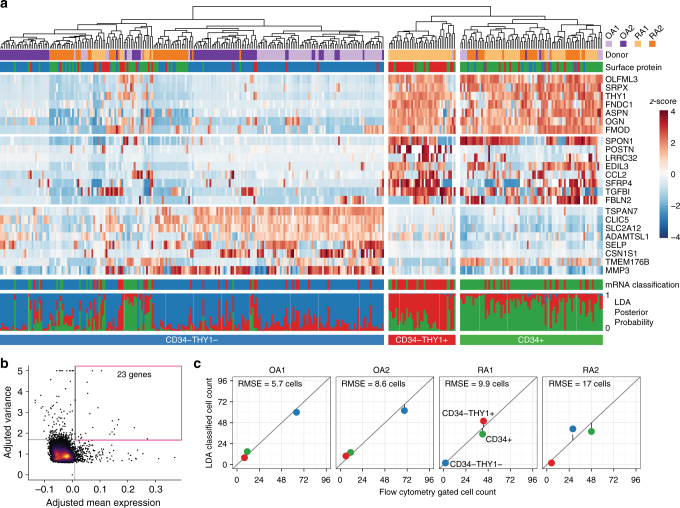
Unbiased clustering of fibroblasts by single-cell RNA-seq. **a** Hierarchical clustering is concordant with our surface protein gating. The first track indicates which donor the cell came from. The second track indicates which cell sorting gate the cell falls into, by surface protein expression. The heatmap indicates mRNA expression, scaled across cells to reveal variation between cells. Below the heatmap, the first track shows linear discriminant analysis (LDA) classification by mRNA expression of 968 genes. The last track shows posterior probabilities of LDA classification. **b** For the heatmap, we selected 23 genes with greatest 1% mean and greatest 1% variance in expression levels. **c** The number of cells in each subset as determined by surface protein levels (*x*-axis) and by LDA classification (*y*-axis) is consistent. RMSE, root mean squared error

Next, since our observation from gated fibroblast populations may be potentially biased by our a priori selection of surface markers, we performed single-cell mRNA sequencing to obtain an unbiased characterization of transcriptional heterogeneity in synovial fibroblasts. Single fibroblasts from four additional donors (two RA and two OA) were isolated by flow cytometry (PTPRC^–^ GYPA^–^ PECAM1^–^ PDPN^+^), followed by single-cell library generation (Illumina Smart-Seq2) (Supplementary Fig. 1C). The average sequence depth was 5.4M fragments per cell, resulting in detection of an average of 8,842 genes per cell with at least 1 transcript per million (TPM) (Supplementary Fig. 4). Three hundred and thirty-seven cells with at least 5,000 detected genes were used for single-cell differential expression to estimate error models for each cell, normalize expression values, remove aspects of variation due to library complexity, and correct for batch effects across donors^18^. Simultaneous surface protein markers were obtained on each single cell by flow cytometry at the time of single-cell sorting. Remarkably, unbiased subsets (defined by hierarchical clustering of single cells with 23 genes that show high mean and variance across cells) were concordant with the three subsets defined by protein levels of CD34 and THY1 surface markers (*P* < 10^−4^; Permutation test) (Fig. 2a, b).

Next, we assessed whether the bulk and single-cell RNA-seq profiles were marking similar cellular subpopulations. We trained a linear discriminant analysis (LDA) classifier on 968 genes in the bulk RNA-seq data and predicted the classes of single cells (see Methods). The LDA classifier produced a probability of belonging to each class (CD34^–^THY1^–^, CD34^–^THY1^+^, and CD34^+^). The classifications were confident (median probability 0.8) and consistent with the three subsets (Fig. 2a). We compared mRNA classification with flow cytometric protein marker data recorded at the time of unbiased single-cell sorting and found concordant cell identities and proportions (Fig. 2c, Supplementary Fig. 5). These single-cell RNA-seq results provide an unbiased and independent validation of the three major subsets defined with bulk transcriptomics of samples gated by protein surface markers.

### Fibroblast subsets localize to specific regions in the synovium

**Fig. 3:**
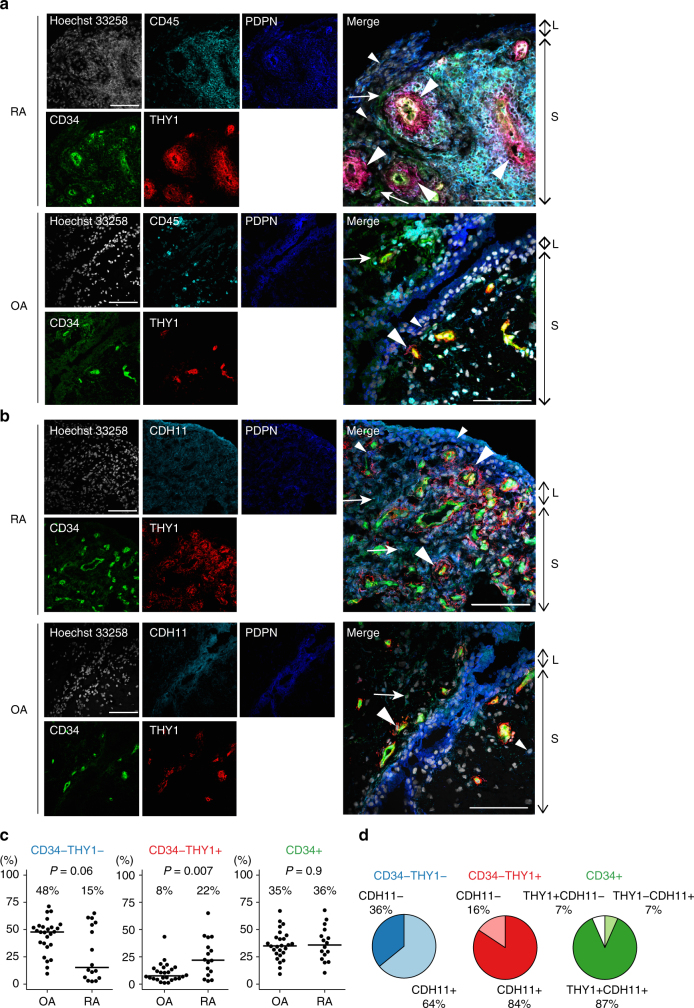
Anatomical localization and proportions of fibroblast subsets. **a** Anatomical localization of fibroblast subsets and leukocytes in RA and OA synovial tissue. Hoechst 33258: white, CD45: cyan, PDPN: blue, CD34: green, THY1: red. Small arrowheads: CD34^–^THY1^–^ fibroblasts. Big arrowheads: CD34^–^THY1^+^ fibroblasts. Arrows: CD34^+^ fibroblasts. Scale = 100 µm. **b** Expression of fibroblast subset markers in RA and OA synovial tissue. Hoechst 33258: white, CDH11: cyan, PDPN: blue, CD34: green, THY1: red. **c** Proportions of fibroblast subsets in synovial tissue in OA (*n* = 26) and RA (*n* = 16) evaluated by flow cytometry. *P* values from the Wilcoxon's rank-sum test. **d** Proportions of CDH11^+^ cells in CD34^–^THY1^–^ fibroblasts, CD34^–^THY1^+^ fibroblasts, and CD34^+^ fibroblasts. L lining area, S sublining area

Since fibroblast identity is highly dependent on the microenvironment, we sought to determine whether anatomical localization of fibroblast subsets could contribute to transcriptomic heterogeneity in OA and RA synovial tissue (Fig. 3a, b, and Supplementary Fig. 6). Interestingly, CD34^–^THY1^+^ fibroblasts in RA form a discrete perivascular zone surrounding capillary structures in the deep sublining layer of the synovium, especially near accumulations of lymphocytes. In contrast, CD34^–^THY1^+^ fibroblasts comprise a thin layer with fewer cells surrounding blood vessels in OA (Fig. 3a). CD34^+^ fibroblasts were observed in both superficial lining and deeper sublining areas of the synovium. CD34^–^THY1^–^ fibroblasts were mostly observed in lining area. The expression of CDH11 were observed in the majority of fibroblasts, with highest expression observed in the lining layer (Fig. 3b, Supplementary Fig. 7).

### Fibroblast subset proportions are altered in RA

We hypothesized that if pathological fibroblast subsets exist, then some subsets might be more or less abundant in RA relative to OA synovial tissues. Indeed, proportions of fibroblast subsets defined by flow cytometry with protein surface markers were different between RA synovial tissue (*n* = 16) and OA (*n* = 26) (Fig. 3c, Supplementary Table 1). To account for testing three subsets of fibroblasts, we consider a *P* value of 0.05/3 = 0.017 to be significant. The proportion of CD34^–^THY1^+^ fibroblasts comprised a median of 22% of total fibroblasts in RA compared to 8% in OA (odds ratio (OR) = 3 (95% confidence interval (CI): 1.33–6.48), *P* = 0.007 (Wilcoxon's rank-sum test)). By contrast, CD34^–^THY1^–^ cells were less abundant in RA at 15% compared to 48% in OA (OR = 0.48 (95% CI: 0.23–1.03), *P* = 0.06 (Wilcoxon's rank-sum test)). Within 12 RA samples, 7 samples were obtained from swollen joints according to a rheumatologist’s assessment, and 5 samples were from non-swollen joints (Supplementary Fig. 8). Although we acknowledge that the presence or absence of swelling is not a robust parameter to reflect joint inflammation, we found that the swollen joints had fewer CD34^–^THY1^–^ (*P* = 0.02), more CD34^–^THY1^+^ (*P* = 0.09), and more CD34^+^ fibroblasts (*P* = 0.01 (Wilcoxon's rank-sum test)). Notably, most cells in the expanded CD34^–^THY1^+^ population in RA also were positive for surface protein levels of CDH11 (median 84%), and CDH11 was also expressed on the other fibroblast subsets including CD34^–^THY1^–^ (median 64%) and CD34^+^ (median 94%) cells (Fig. 3d). These results suggest that synovial fibroblast subpopulation proportions are closely related to disease type and activity.

**Fig. 4:**
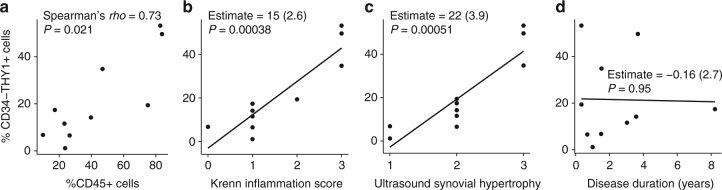
Correlation between proportion of fibroblast subsets and synovial inflammation. **a** The Spearman's correlation between the proportion of CD34^–^THY1^+^ fibroblasts in total fibroblasts and the proportion of CD45^+^ cells in total live cells in synovial tissue biopsies from RA knee joints. **b**–**d** The linear correlation coefficient between the proportion of CD34^–^THY1^+^ fibroblasts and Krenn inflammation score (**b**), ultrasound synovial hypertrophy score (**c**), and disease duration (years) (**d**). Proportion of cells were evaluated by flow cytometry. Plots **b**–**d** show linear model coefficient estimates and *t*test *P* values

We note that all OA samples were taken from the knee, while RA samples included those from the knee (*n* = 8) as well as other smaller joints (*n* = 8) (Supplementary Table 1). To confirm that the altered proportion of fibroblast subsets in RA reflects the level of tissue inflammation, rather than joint location of origin, we collected 10 independent RA synovial tissue biopsies from only knee joints and examined the proportion of fibroblast subsets and infiltrated leukocytes by flow cytometry (Supplementary Table 2). We selected only samples with synovial hypertrophy on ultrasound images. Though our sample size (*n* = 10) was limited, we saw that the proportion of CD34^–^THY1^+^ fibroblasts is positively correlated with the proportion of infiltrated leukocytes by flow cytometry (Fig. 4a). In addition, the proportion of CD34^–^THY1^+^ fibroblasts correlated with both histological synovitis and synovial hypertrophy assessed by ultrasound (Fig. 4b, c). These results indicate that the altered proportion of fibroblast subsets in RA reflects tissue inflammation at both the molecular and clinical level. In contrast, the correlation between the proportion of CD34^–^THY1^+^ fibroblasts and disease duration was not observed, suggesting that the altered proportion of fibroblast subsets is not a secondary effect of chronic tissue damage (Fig. 4d).

### Transcriptomics predicts distinct fibroblast functions

**Fig. 5:**
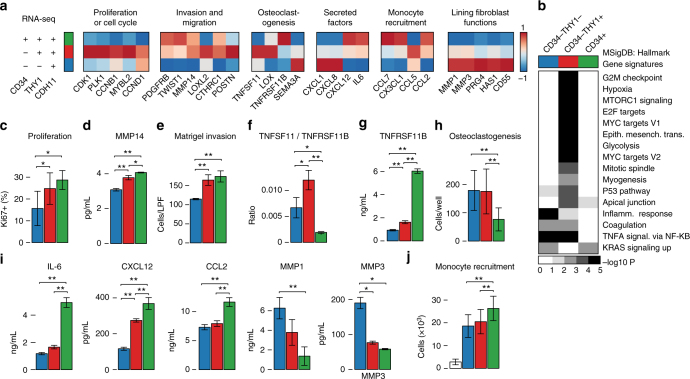
Functional differences between fibroblast subsets. **a** Gene expression pattern of key effector molecules to predict the function of fibroblast subsets. **b** Gene set enrichment analysis with RNA-seq samples and MSigDB Hallmark Gene Signatures. Terms with <5% FDR are displayed. **c** The proportion of Ki67-positive cells in freshly isolated synovial fibroblast subsets. **d** Protein expression of MMP14 in the cell lysates of expanded fibroblast subsets in the presence of 1 ng/mL TNF-α. **e** The number of migrated and invaded cells in transwell matrix invasion assay. Fifty nanogram per mL of PDGFBB was used to promote migration and invasion. **f** The ratio of TNFSF11 (RANKL) and TNFRSF11B (OPG) in mRNA level in expanded fibroblast subsets. **g** Protein expression of TNFRSF11B in the supernatant of expanded fibroblast subsets. **h** Number of osteoclasts developed in the co-culture of osteoclast progenitors with fibroblast subsets in the presence of 20 ng/mL M-CSF and 5 ng/mL RANKL. **i** Protein expression of cytokines and MMPs in the supernatant of expanded fibroblast subsets in the presence of 1 ng/mL TNF-α. **j** The number of migrated monocytes in monocyte recruitment assay using supernatant of cultured fibroblast subsets. In bar graphs, blue, red and green color coding corresponds to the fibroblast subsets shown in **a**. White bar in **j** indicates culture media without fibroblasts. Bars show means and error bars show standard deviations. One-way ANOVA, Tukey's post hoc: *P < 0.05; ** *P* < 0.005

We hypothesize that several key effector molecules differentially expressed by fibroblast subsets may represent key transcriptional modules that could predict distinct cellular functions among fibroblast subsets (Fig. 5a). To test whether these transcriptional modules correlate with cellular functions in vitro, fibroblast subsets were isolated and maintained as primary cells and the predicted function associated with each module was examined.

First, gene set enrichment analysis revealed significant enrichment of mitotic and proliferative genes among the CD34^–^THY1^+^ subset (Fig. 5b). Consistent with an actively proliferative state, both CD34^–^THY1^+^ and CD34^+^ populations exhibited significantly higher proportion of Ki67-positive cells compared to CD34^–^THY1^–^ fibroblasts (Fig. 5c). Next, we found that genes implicated in fibroblast migratory response, including *CTHRC1*, *TWIST1*, *POSTN*, *LOXL2*, *PDGFRB*, and *MMP14*^19–23^, were elevated in CD34^–^THY1^+^ and CD34^+^ fibroblasts (Fig. 5a, d), suggesting elevated migratory and invasive behavior. Indeed, CD34^–^THY1^+^ and CD34^+^ fibroblasts exhibited enhanced in vitro invasion and migration in response to platelet derived growth factor BB (PDGFBB) in a transwell matrix invasion assay (Fig. 5e).

In RA, an important pathogenic effector function of synovial fibroblasts is modulation of osteoclastogenesis, a process predominantly driven by TNFSF11 (also known as receptor activator of nuclear factor-κΒ ligand (RANKL)) and opposed by TNFRSF11B (also known as osteoprotegerin (OPG)), which is a decoy receptor for TNFSF11^24–27^. Analysis of genes related to osteoclastogenesis revealed high expression of *TNFSF11* and low expression of *TNFRSF11B* in CD34^–^THY1^+^ fibroblasts (Fig. 5a, f). Low expression of *TNFRSF11B* in CD34^–^THY1^–^ fibroblasts and CD34^–^THY1^+^ fibroblasts was confirmed by direct protein measurement from supernatant of fibroblast subset (Fig. 5g). In support of their role in promoting osteoclast differentiation, co-culture of CD34^–^THY1^+^ or CD34^–^THY1^–^ fibroblasts with peripheral blood monocytes led to increased number of tartrate-resistant acid phosphatase (TRAP)-positive osteoclastic cells in vitro (Fig. 5h).

Chemokine secretion and recruitment of leukocytes to inflamed tissue is a major function of synovial fibroblasts^2,4^. CD34^+^ fibroblasts have a transcriptomic profile characterized by the expression of inflammatory cytokine genes *IL6*, *CXCL12*, and *CCL2* (Fig. 5a). We also confirmed that CD34+ fibroblasts secrete large amounts of proteins IL-6, CXCL12, and CCL2 when stimulated with tumor necrosis factor (TNF) in vitro (Fig. 5i). Furthermore, their enhanced secretory phenotype was reflected by their ability to recruit significantly higher number of peripheral blood monocytes in a transwell leukocyte recruitment assay (Fig. 5j). Taken together, these results suggest a greater role for CD34^+^ fibroblasts in monocyte recruitment in inflamed synovial tissue.

Finally, we examined the expression of genes that have been reported to be highly expressed in lining fibroblasts. We found that CD34^–^THY1^–^ fibroblasts express high levels of genes *MMP1*, *MMP3*, *PRG4*, *HAS1*, and *CD55* (Fig. 5a). High expression levels of proteins MMP1 and MMP3 in CD34^–^THY1^–^ fibroblasts were also confirmed (Fig. 5i). These results indicate that at least some of the CD34^–^THY1^–^ fibroblasts are lining fibroblasts.

## Discussion

This study identifies subsets of fibroblasts in fresh human synovium, including a distinct subset of PDPN^+^CD34^–^THY1^+^ fibroblasts that is expanded in RA and may be pathogenic. These cells are enriched around blood vessels in RA synovium, and their expression profile reveals potential pathogenic roles in matrix invasion, immune cell recruitment, and osteoclastogenesis. We note that almost all of these cells are positive for protein CDH11, which we have previously shown to be associated with pathological behavior of fibroblasts in in vitro studies and RA mouse models^3^.

Expansion of fibroblasts is a dynamic component of RA synovitis. An increase in synovial lining fibroblasts was noted previously to correlate with disease activity score in 28 joints (DAS28), disease duration, and the level of macrophage infiltration^28^. Expansion of synovial sublining fibroblasts is also observed in RA, but no previous literature reports significant correlation between sublining fibroblasts and other clinical or pathological findings, except for a negative correlation with DAS28 in one study^28^. Here, by separating fibroblasts into subsets based on the expression patterns of multiple markers, we found that the increase of CD34^–^THY1^+^ fibroblasts around blood vessels in the sublining area is a dominant change in fibroblasts in RA synovium. Moreover, this expansion distinguishes RA from OA, reflects RA disease activity, and correlates with immune cell infiltration in the synovium. Previous studies have shown that mesenchymal CDH11 determines adhesion between fibroblasts, increases their migration and invasion, and synergizes in the activation of fibroblasts to produce MMPs, cytokines, and chemokines^3,29,30^. Since a large majority of the expanded fibroblast population expresses this cadherin, it also may contribute to their pathologic behavior in RA (Fig. 3d).

High *TNFSF11* and low *TNFRSF11B* gene expression in freshly isolated CD34^–^THY1^+^ fibroblasts suggest that the increased number of CD34^–^THY1^+^ fibroblasts in RA is involved in the increased bone destruction in RA. However, CD34^–^THY1^–^ and CD34^–^THY1^+^ fibroblasts did not differ in their abilities to promote osteoclastogenesis in vitro. We believe that the in vivo synovial environment might be required for CD34^–^THY1^+^ fibroblasts to fully function as stromal cells that promote osteoclastogenesis. This subset may also be responsible for the accumulation of lymphocytes in RA synovial tissue, since TNFSF11 is involved in T cell trafficking in autoimmune inflammation^31^. In addition, reduced proportions of CD34^–^THY1^–^ fibroblasts may explain the decreased bone formation activity in RA, since they also express *BMP-6*, known to promote osteoblastic bone formation^32^. Taken together, the altered proportions of fibroblast subsets in RA may cause aberrant homeostasis in the joint that supports joint destruction.

We noticed that differential expression of a number of genes among freshly isolated fibroblast subsets were not retained ex vivo or did not translate to functional differences in vitro. These differences between fresh cells and cultured cells are likely due to the effects of the culture conditions and loss of local factors in the microenvironment. Identification of key signaling pathways relevant in maintaining fibroblast subset identity in in vitro culture would greatly facilitate in-depth functional analysis and further shed light on the biological significance of fibroblast heterogeneity.

We also recognize that the response to enzymatic digestion might be different between fibroblast subsets, and this could lead to differences in gene expression profiles. However, all samples were digested with the same procedure, and we observed similar differences in gene expression across three types of expression data, including microarray and RNA-seq assays of sorted fibroblasts as well as single-cell RNA-seq of freshly isolated single cells. Further, in vitro assays with expanded cells showed functional differences consistent with gene expression data. In previous studies, others have now shown that cells isolated from organ tissues retain distinct transcriptional profiles^33,34^. Thus, the analysis of freshly isolated cells from affected organs in diseases is useful to identify pathogenic subsets of cells and their functions in the pathogenesis.

Our gene expression data is limited to synovial specimens from the latest stages of RA and therefore represent changes in chronic late-stage RA. Gene expression analysis of synovial biopsy specimens from early stage of RA are needed to learn more about pathogenesis at early stages of disease.

There is a real need for strategies to define fibroblast heterogeneity and pathogenic fibroblast populations in order to understand the complex nature of tissue pathology and the role of tissue resident cells in end-organ damage. Our approach involves an integrative analysis of cell surface markers, bulk transcriptomes, single-cell transcriptomics, and histological imaging of human tissues that identified a disease-related fibroblast subpopulation that may ultimately serve as a specific target for therapy. As there are no approved drugs that directly target fibroblasts, identifying pathogenic fibroblast subsets may reveal therapeutic targets broadly applicable across a range of diseases. In RA, targeting fibroblast subsets might complement anti-inflammatory therapies that target leukocytes and their cytokines^4^.

Additional clinical studies are required to understand how the alteration of these subsets are involved in a variety of clinical contexts in RA including the severity of disease activities, prognosis of joint destruction, and response to therapies. We anticipate that this study will serve as a roadmap for these studies, and may serve as a template for future studies to identify pathogenic subsets of tissue cells in other human diseases.

## Methods

### Patient recruitment and isolation of synovial cells

We obtained synovial tissue from joint replacement surgeries, synovectomy surgeries, or synovial biopsies of OA or RA patients with appropriate informed consent as required. The study protocols are Institutional Review Board approved at Partners HealthCare, Hospital for Special Surgery, and the University of Birmingham Local Ethical Review Committee. We collected all available RA samples for the studies. OA samples were collected at random from joint replacement surgeries without any bias. We modified previously described protocols to isolate synovial cells^35,36^. Briefly, we obtained tissue immediately after the surgeries. We removed bone and adipose tissues with scissors. We cut synovial tissues into small pieces, and then subjected these pieces to enzymatic digestion. For microarray analysis, low input bulk RNA-seq and in vitro assays, we digested tissues with 4 mg/mL collagenase type 4 (Worthington, NJ, USA), 0.8 mg/mL dispase II, 0.1 mg/mL DNaseI (Roche) in Dulbecco’s modified Eagle’s medium (DMEM) at 37 °C. After 15 min, we collected the supernatant and replaced with fresh enzyme mix. We repeated these procedures every 15 min for total 1 h. For the analysis of synovial biopsy samples, we digested the tissues with 0.05 mg/mL Liberase TM (Roche) and 0.04 mg/mL DNaseI at 37 °C for 30 min. For single-cell RNA-seq, we digested tissues with 0.2 mg/mL Liberase TL (Roche), 0.1 mg/mL DNaseI in RPMI at 37 °C for 20 min to minimize the cleavage of surface markers of lymphocytes during the enzymatic digestion. After lysing red blood cells with ACK-lysing buffer, we stained cells with antibodies, and sorted by FACSAria Fusion (BD) with 100 μm nozzle at 20 psi. For the analysis with microarray and low input RNA-seq, the cells were sorted into 2% fetal bovine serum (FBS) HBS+ buffer, spun down, and lysed with TRIzol (Invitrogen). We extracted RNA and cleaned up by RNeasy micro kit (Qiagen) with DNaseI treatment. For single-cell RNA-seq, the cells were stained with antibodies and directly sorted into 5 μl of TCL buffer (Qiagen) with 1% β-mercaptoethanol (Sigma) in 96-well plates.

### Antibodies and reagents

The following antibodies and reagents were used for the analysis of synovial cells with flow cytometry and cell sorting: anti-CD45-APC-H7 (2D1, BD Pharmingen), anti-CD235a-APC-Alexa Fluor750 (11E4B-7-6(KC16), Beckman Coulter), anti-CD31-PE-Cyanine7 (WM-59, eBioscience), anti-CD146-BV450 (P1H12, BD Horizon), anti-CD34-PE (4H11, eBioscience), anti-PDPN-PerCP-eFluor710 (NZ-1.3, eBioscience), anti-THY1-FITC (5E10, BD Pharmingen), anti-cadherin-11-biotin (23C6), human TruStain FcX (BioLegend), streptavidin-APC (Jackson ImmunoResearch), Live/Dead fixable aqua dead cell stain kits (Molecular Probes). For immunofluorescence staining of synovial tissue, following antibodies and reagents were used: anti-CD45 (135-4C5, AbD Serotec), anti-CD34 (EP373Y, Abcam), anti-PDPN (NZ-1.3, eBioscience), anti-THY1 (F15-42-1, Merck Millipore, and clone Thy-1A1, R&D Systems), anti-cadherin-11-Biotin (23C6), anti-Ki67 (16A8, BioLegend), anti-mouse IgG1-FITC (Southern Biotech), anti-mouse IgG2a FITC (Southern Biotech), anti-mouse IgG2b-Alexa Fluor 647 (Life Technologies), anti-rat IgG-Alexa Fluor 594 (Life Technologies), anti-rat IgG-Alexa Fluor 647, anti-rabbit IgG-Alexa Fluor 546 (Life Technologies), Hoechst 33258 (Life Technologies), and anti-FITC Alexa Fluor 488 (Life Technologies).

### Gene expression microarrays

We evaluated the integrity of RNA with Bioanalyzer or by Tapestation (Agilent). We used only RNA with more than RNA integrity number score of 7. We prepared complementary DNA (cDNA) from 38.1 g total RNA using Ovation Pico WTA (NuGEN), followed by labeling 5 μg cDNA using Biotin Module (Nugen). We assayed gene expression using the GeneChip Human 2.0 ST microarray (Affymetrix). We normalized expression by Robust Multiarray Averaging (RMA). We identified and removed two outlier arrays by PCA. We assigned probes to Entrez Gene IDs using Ensembl BioMart on 17 March 2015. In the instance where there were multiple probesets assigned to a single gene, we averaged them to obtain a single gene value.

### RNA-seq library preparation and sequencing

We used 1,250 cells per sample for library preparation. We prepared sequencing libraries using the Smart-Seq2 protocol. We pooled and sequenced libraries were pooled and sequenced with the Illumina HiSeq 2500 to a depth of 8–14M reads per library.

### Single-cell RNA-seq

We assayed 384 fibroblasts from four donors, two with OA and two with RA. For each donor, we collected fresh synovial tissue, isolated synovial cells by enzymatic digestion, and stained with antibodies against CD45, CD235a, CD31, CD146, PDPN, CD34, THY1, and CDH11. We sorted 96 single CD45^–^CD235a^–^CD31^–^PDPN^+^ cells by FACSAria Fusion (BD), and assayed mRNA expression with the Smart-Seq2 protocol^37^. Single-cell libraries were also prepared with the same protocol, and we aimed to sequence to a depth of 200K–12M reads per library^38^. On average, we sequenced 5.4M fragments and detected 8,153 genes per cell with at least 1 TPM. We discarded 47 cells (12%) with fewer than 5,000 genes detected from further analysis.

### RNA-seq gene expression quantification

We quantified cDNAs on canonical chromosomes in Ensembl release 83 with Kallisto v0.42.4^39^ in TPM and summed to get gene-level expression values. We quantified gene expression the same way for bulk and single-cell RNA-seq samples. For differential expression analysis, hierarchical clustering, and PCA, we log (base 2)-transformed TPM values.

### Lineage marker analysis

We selected lineage markers for fibroblast, endothelial, and hematopoietic cells^12^ and checked their expression levels to confirm that our samples are from the fibroblast lineage (Supplementary Fig. 1).

### Differential expression analysis with microarrays

We used the R package limma to assess differential expression analysis on RMA normalized expression values^40^. Before performing differential expression analysis, donor-specific variation was regressed out by obtaining the residuals from linear models. In order to regress out effects, we modeled each gene as a linear combination of donor-specific effects. After using ordinary least squares to fit the donor-specific effects, we calculated residuals for each gene. These residuals were then used for differential expression analysis, hierarchical clustering, and PCA. We expected differences in gene expression between RA and OA. However, we lacked power to see these differences within fibroblast subsets.

### Differential expression analysis with RNA-seq

RNA-seq data was analyzed the same way as the microarray data, starting with log 2-transformed TPM expression values. Donor-specific variation was similarly regressed out before differential expression analysis, hierarchical clustering, and PCA.

### Gene set enrichment analysis

Terms from MSigDB Hallmark Gene Signatures^41^ were used for enrichment analysis with LIGER^42^. We tested gene sets for enrichment with differential expression between the three major subsets in order to assess how they might differ from each other in terms of molecular pathways. MSigDB hallmark pathways enriched with differential expression signal for CD34^–^THY1^+^ population, expanded in RA joints, include “epithelial-to-mesenchymal transition,” “hypoxia,” and “glycolysis.”^42^

### Principal components analysis

After gene selection by analysis of variance (ANOVA) or differential expression analysis, we scaled each sample and then scaled each gene across the samples to obtain a specificity of the gene to each sample. Next, we used the prcomp function in R to perform PCA with centered and scaled log 2 expression values.

### Linear discriminant analysis

We checked if transcriptional profiles of single cells are similar to profiles of the three major subsets we defined by bulk transcriptomics. First, we selected 1,171 genes with significant (5% FDR) differential expression between any pair of the three subsets in bulk RNA-seq data. We selected a subset of 968 genes with high expression in single cells (mean log 2(TPM) > log 2(10)). We used the bulk RNA-seq data to train an LDA model with these genes, and then classified each single cell’s expression profile to predict each single cell’s identity. The confidence of each classification is represented by a posterior probability.

### Histological analysis

RA synovial tissues were obtained by biopsies from RA patients in the BEACON Birmingham early arthritis cohort, which is an early arthritis cohort recruiting patients with new onset arthritis prior to treatment with disease-modifying antirheumatic drugs. Synovial tissues for staining were frozen in OCT compound. Sections were made in 6 μm thickness, fixed with acetone, and frozen prior to use. Slides were rehydrated in phosphate-bufferred saline (PBS), blocked with 10% normal goat or horse serum in PBS for 10 min, and then incubated with primary antibodies, followed by secondary antibodies. Slides were mounted using ProLong Diamond (Life Technologies), and imaged using a Zeiss LSM 780 or 800 confocal microscope. Images were processed using Zen Black and Zen lite (Zeiss). Representative images were shown. Synovial tissues for histological analysis and hematoxylin and eosin staining were fixed in formaldehyde, then mounted in paraffin, sectioned, and stained by the Hospital Pathology service.

### Histological evaluation of inflammatory infiltrate

Hematoxylin-stained and eosin-stained sections of knee synovial biopsy samples were examined histologically for the severity of inflammatory infiltrate using the inflammatory component of the Krenn synovitis score^43^. Inflammatory infiltrates were graded from 0 to 3 (0 = no inflammatory infiltrate, 1 = few mostly perivascular situated lymphocytes or plasma cells, 2 = numerous lymphocytes or plasma cells sometimes forming follicle-like aggregates, and 3 = dense band-like inflammatory infiltrate or numerous large follicle-like aggregates). The tissues were graded in a blinded manner by two trained individuals and then provided consensus.

### Clinical evaluation of synovitis by ultrasound

The joint to be biopsied was assessed using ultrasound immediately prior to the procedure using a Siemens Acuson Antares scanner (Siemens PLC, Bracknell, UK) and multifrequency (5–13 MHz) linear array transducers. Synovitis and power Doppler (PD) positivity were defined using consensus OMERACT definitions^44^. Gray-scale synovial hypertrophy and PD ultrasound variables were graded on 0–3 semi-quantitative scales as previously reported^45^.

### Cell culture

We sorted CD34^–^THY1^–^ fibroblasts, CD34^–^THY1^+^CDH11^+^ fibroblasts, and CD34^+^THY1^+^CDH11^+^ fibroblasts, and cultured them in DMEM supplemented with 10% FBS (Gemini), 2 mM l-glutamine, antibiotics (penicillin and streptomycin), and essential and nonessential amino acids (Life Technologies). The cells were expanded for 3–20 days for assays in vitro. The cells with one or two passages were used. The cells were cultured in the presence or absence of 1 ng/mL of TNF-α (R and D) for 24 h for enzyme-linked immunosorbent assay (ELISA) of IL-6, CXCL12, MMP1, MMP3, and MMP14, or for 72 h for quantitative real-time PCR (qPCR) of TNFSF11 and TNFRSF11B.

### ELISA

The levels of IL-6, CXCL12, MMP1, MMP3 and TNFRSF11B in the supernatant or the levels of MMP-14 in the cell lysate were evaluated by ELISA kit as described in manufacturer’s instructions (Duo Set, R and D).

### Quantitative real-time PCR

cDNA was synthesized with QuantiTect Reverse Transcription kit (Qiagen). qPCR was performed with Brilliant III Ultra-Fast SYBR Green qPCR master mix (Agilent Technologies) on a Mx3000 (Stratagene). The following primers were used: TNFSF11, forward: 5′-GGA GAG GAA ATC AGC ATC GAG and reverse: 5′-CCA AAC ATC CAG GAA ATA CAT AAC AC; TNFRSF11B, forward: 5′-CAA CAC AGC TCA CAA GAA CAG and reverse: 5′-GAA GGT GAG GTT AGC ATG TCC; GAPDH, forward: 5′-AAT CCC ATC ACC ATC TTC CAG and reverse; 5′-AAA TGA GCC CCA GCC TTC.

### Osteoclastogenesis assay

Osteoclast progenitors were prepared by culturing peripheral blood mononuclear cell in the presence of 20 ng/mL of macrophage colony-stimulating factor (M-CSF) (PeproTech) in DMEM supplemented with 10% FBS (GE Healthcare), 2 mM l-glutamine, and antibiotics for 5–6 days. Expanded fibroblasts were seeded at 5,000 cells per well in 96-well plates. On the next day, osteoclast progenitors were added at 5,000 cells per well, and were co-cultures with fibroblasts in the presence of 20 ng/mL M-CSF and 5–20 ng/mL RANKL (PeproTech). The media were replaced every 2 days. After 6 days of the co-culture, the cells were fixed by 4% paraformaldehyde. After TRAP staining, TRAP-positive multinucleated cells were counted as osteoclasts.

### Ki67 staining

Disaggregated synovial cells were washed and stained with an extraceullar panel, followed by fixation and permeabilization (Foxp3/Transcription Factor Staining Buffer Set, eBioscience). Ki67-positive cells were quantified by flow cytometry and the percentage of Ki67-positive cells were analyzed and calculated through FlowJo.

### Monocyte recruitment assay

A modified transwell migration assay was used to assess monocyte recruitment by synovial fibroblasts. Supernatants from TNF-α-stimulated synovial fibroblast subsets were collected, diluted 1:1 with RPMI, and placed in the bottom chamber of 24-well plates. A total of 5 × 10^5^ purified human peripheral blood mononuclear cells from healthy donors were resuspended in RPMI and placed in the top chamber of Transwell inserts (Corning). After 3 h, cells that have migrated into the bottom chamber were collected and monocytes were quantified through flow cytometric analysis.

### Transwell matrix invasion assay

Transwell matrix invasion assay was conducted using Corning Matrigel Invasion Chamber with 8.0 μm pore as per the manufacturer’s protocol. Expanded fibroblasts were resuspended in 0.5% bovine serum albumin (BSA)/DMEM and seeded at 15,000 cells per well in upper chamber. Fifty nanogram per mL of PDGFBB in 0.5% BSA/DMEM was used to promote invasion and migration of fibroblasts. After the incubation for 48 h, the non-invading cells were removed from the upper surface of the membrane. The cells on the lower surface of the membrane were stained with Diff-Quick. The number of invaded cells were counted with a microscope.

### Code availability

The authors declare that R source code is available from the corresponding authors upon request.

### Data availability

Microarray and RNA-seq expression data that support the findings of this study have been deposited in GEO with the primary accession code GSE109450.

## Electronic supplementary material

Supplementary Information
