# Supplementary material for: Functionally distinct disease-associated fibroblast subsets in rheumatoid arthritis
Source: Nat Commun. 2018 Feb 23;9:789. doi: 10.1038/s41467-018-02892-y (PMC5824882; doi:10.1038/s41467-018-02892-y)
Supplement: Supplementary file 1 — Supplementary Information [file 41467_2018_2892_MOESM1_ESM.pdf]

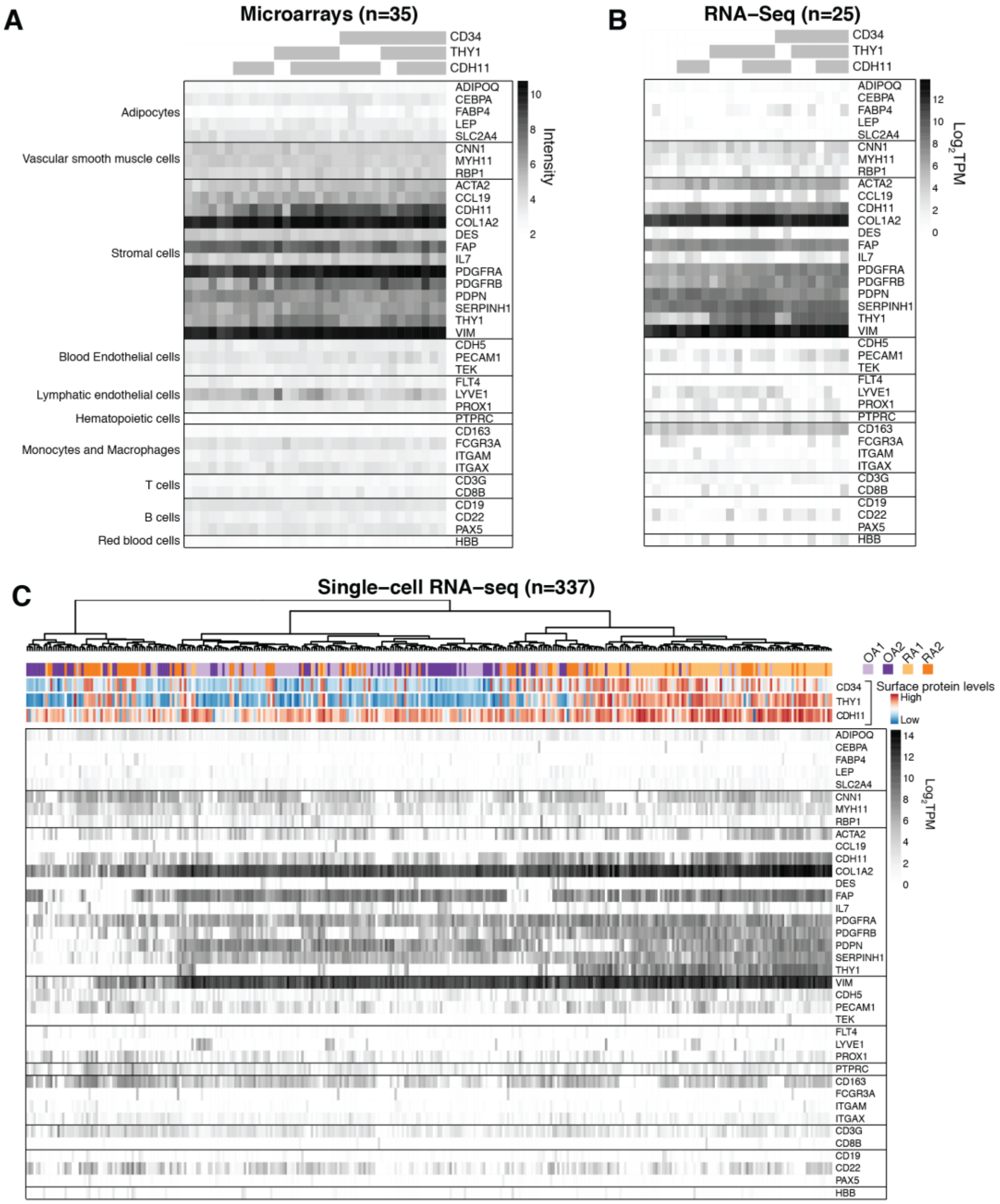

Supplementary Figure 1. Heatmap of expression data for genes characteristic of cell types in synovial tissues. We used fluorescence activated cell sorting (FACS) to enrich samples for mesenchymal stromal cells (MSCs) and deplete other cell types. (**A**) Microarray data (n=32) and (**B**) RNA-seq data (n=25) are annotated with 3 tracks indicating FACS sorting gates for CD34, THY1, and CDH11. (**C**) Single-cell RNA-seq data (n=346) is annotated with FACS data for CD34, THY1, and CHD11, but these cells were not sorted on these markers (see Methods for details). Most samples show high expression of lineage markers for stromal cells, and low expression of lineage markers for adipocytes, vascular smooth muscle cells, blood endothelial cells, lymphatic endothelial cells, hematopoietic cells, monocytes and macrophages, T cells, B cells, and red blood cells.

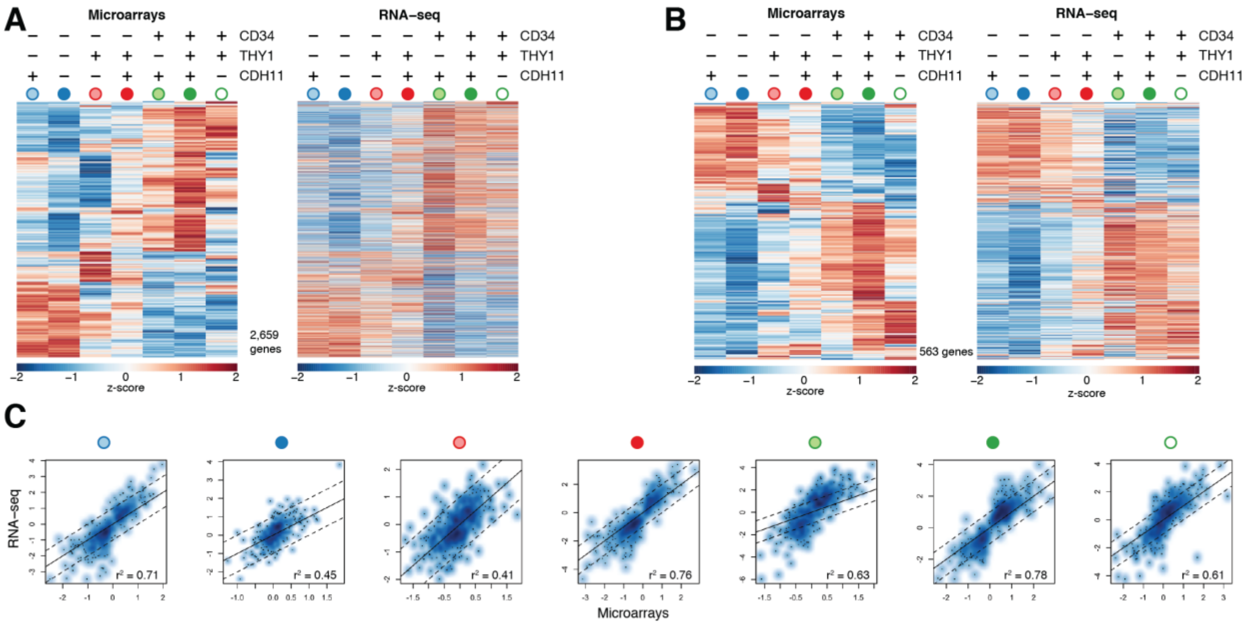

Supplementary Figure 2. Concordance of differential expression between microarrays and low-input RNA-seq. (**A**) ANOVA reveals 2,659 genes that have significant F statistic (1% FDR) across 7 gated subpopulations of cells in the microarray data and are measured in the RNA-seq data. These genes show a consistent pattern across the 7 gated subpopulations. (**B**) 563 genes significant in the RNA-seq data and measured in the microarray data. (**C**) 436 genes significantly differentially expressed in both datasets. The x-axis shows normalized expression in microarray data and y-axis shows normalized expression in RNA-seq data. The variability in correlations might be explained by technical differences between the datasets, differences between donor tissues, or by additional cellular heterogeneity within gated subpopulations. The microarray data represents a summary of tissues from 3 OA and 3 RA donors, while the RNA-seq data is a summary of tissues from 4 RA donors.

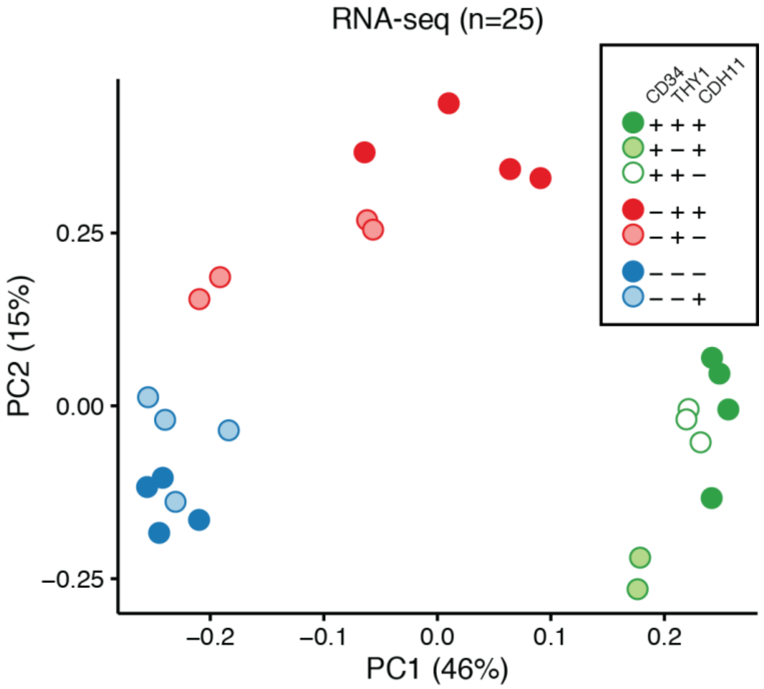

Supplementary Figure 3. Principal component analysis of RNA-seq data. Principal components analysis (PCA) with 671 genes (1% FDR, ANOVA) in microarray data separates the 25 RNA-seq samples into 3 subsets: $CD34^{-}THY1^{-}$, $CD34^{-}THY1^{+}$, and $CD34^{+}$.

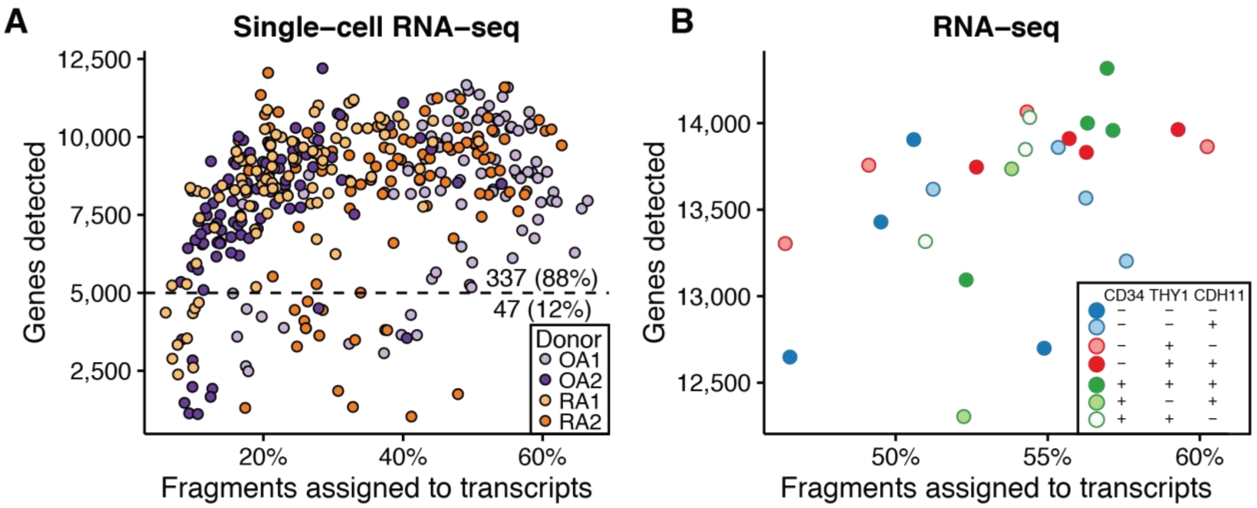

Supplementary Figure 4. Assessing quality of RNA-seq data. The distributions of number of cDNA fragments assigned to mRNA transcripts and the number of genes detected with at least 1 transcript per million (TPM). (**A**) 337 of 384 (88%) cells have 5,000 or more genes detected. We excluded 47 cells with fewer than 5,000 genes detected from analysis. These cells also have low abundance of mitochondrial ribosomal protein genes and stromal cell lineage genes. (**B**) As a point of reference, 25 bulk RNA-seq samples are all high quality in terms of fragments assigned to transcripts and number of genes detected.

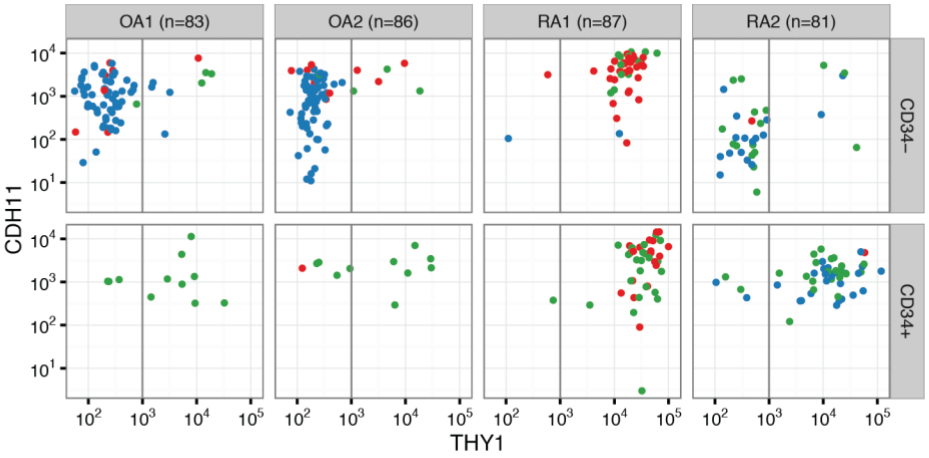

Supplementary Figure 5. Agreement between protein gating and LDA classification based on single cell RNA-seq. Single cells were colored by mRNA classification and displayed as they would have been gated by protein levels. For example, $CD34^{-}THY1^{+}$ cells are expected to be red if the two methods are in perfect agreement. Agreement between protein gating and LDA classification for OA1, OA2, RA1, RA2 is 82%, 87%, 62%, 44%, respectively. Protein level boundaries were set at 600 for CD34 and 1,000 for THY1.

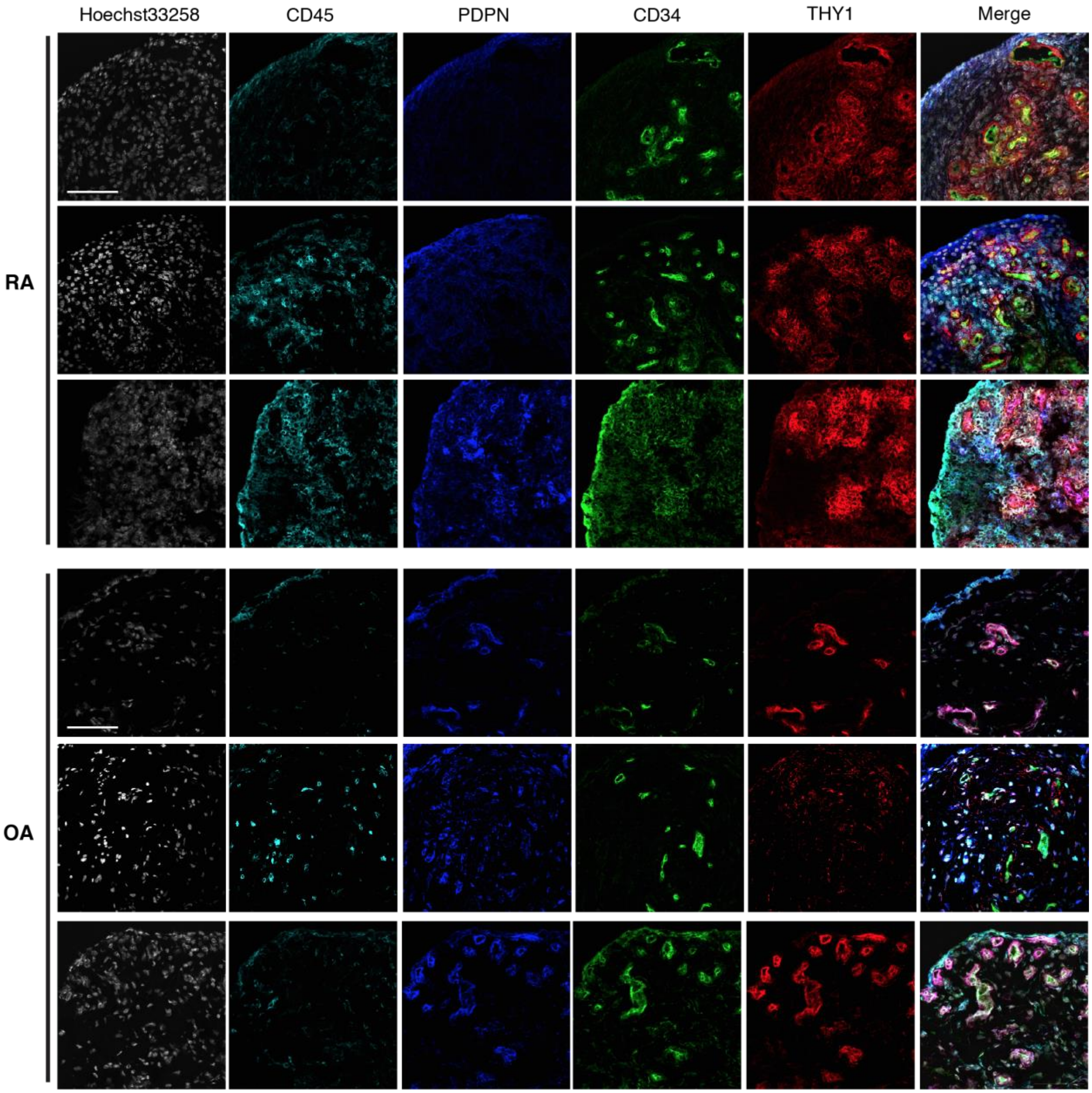

Supplementary Figure 6. Anatomical localization of fibroblast subsets and leukocytes in RA and OA synovial tissue. Hoechst 33258: White, CD45: Cyan, PDPN: Blue, CD34: Green, THY1: Red. Scale = 100 µm.

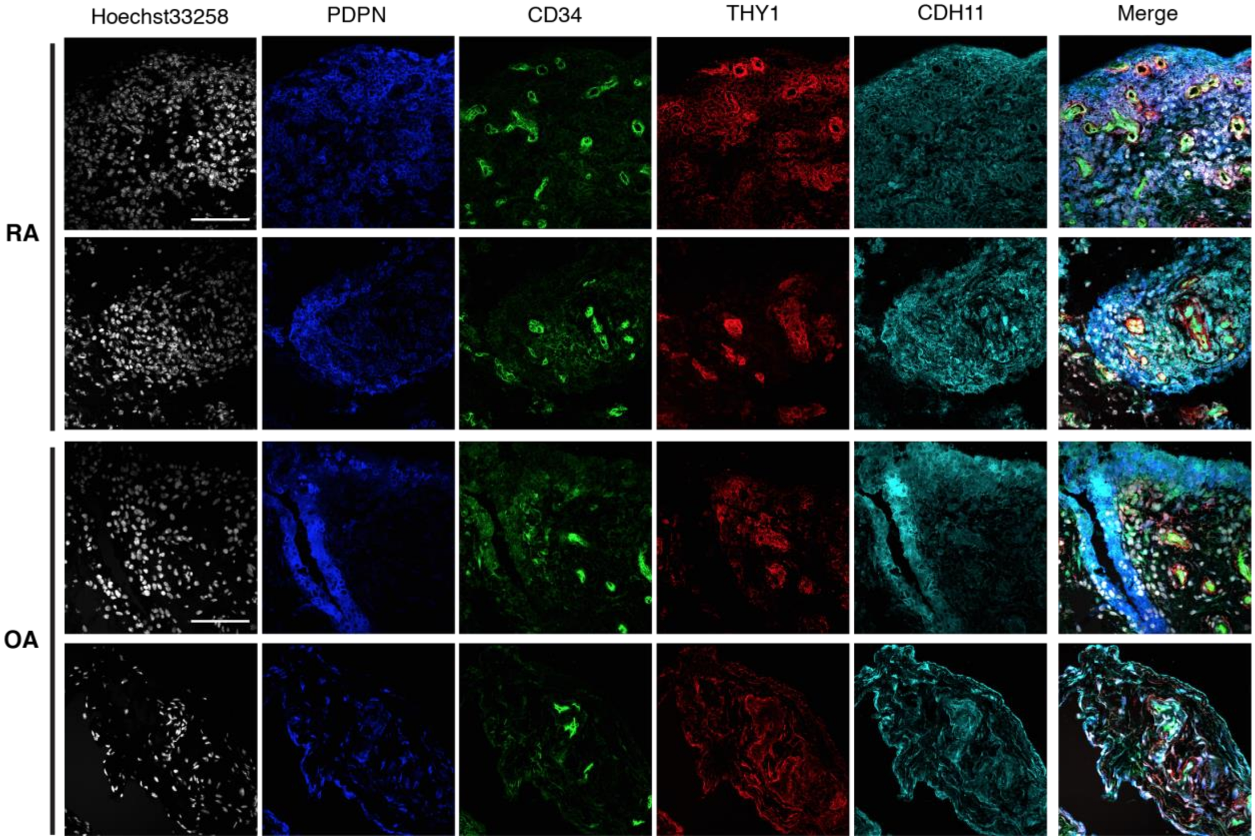

Supplementary Figure 7. Expression of fibroblast subset markers in RA and OA synovial tissue. Hoechst 33258: White, CDH11: Cyan, PDPN: Blue, CD34: Green, THY1: Red. Scale = 100 µm.

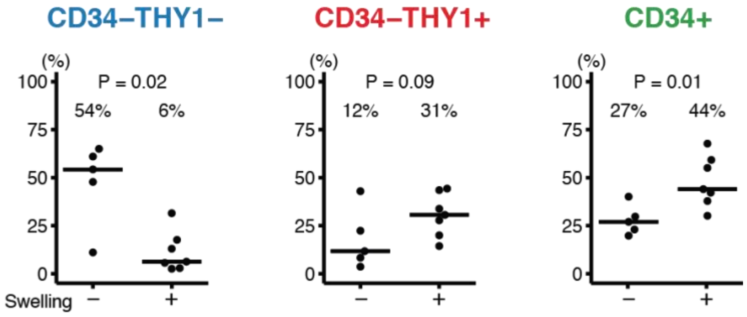

Supplementary Figure 8. Proportion of fibroblast subsets in swollen and non-swollen joints in RA patients. Proportion of cells were evaluated by flow cytometry. Swollen joint: n=7. Non-swollen joint: n=5.

Disease	OA	RA
Number	26	16
Age (years ± SD)	70 ± 9	61 ± 14
Female (%)	54	75
Joint (%)		
Knee	100	50.0
Wrist		25.0
MCP		6.25
MTP		6.25
Elbow		12.5
RF or ACPA (%)		
Positive		56
Negative		6
Unknown		38
CRP (mg/l)		13 ± 22.7
Methotrexate (%)		56.3
Other synthetic (%)		18.8
Biologic (%)		68.8
Glucocorticoid (%)		31.3

Supplementary Table 1. Clinical characteristics of evaluated patients with flow cytometry. MCP; metacarpophalangeal joints, MTP; metatarsophalangeal joint, RF; rheumatoid factor, ACPA; anti-citrullinated protein antibody, CRP; C-Reactive Protein. Other synthetic includes leflunomide and sulfasalazine. Biologic includes TNF inhibitors, abatacept, rituximab, tocilizumab and tofacitinib.

	Average ± SD
Number	10
Age (years)	55.4 ± 11.3
Female (%)	40
CDAI	41.7 ± 12.3
RF or ACPA	
Positive (%)	70
Negative (%)	30
CRP (mg/l)	17.0 ± 22.4
ESR (mm/hr)	13.3 ± 9.6
Disease duration (years)	2.4 ± 2.4
Methotrexate (%)	60
Other synthetic (%)	20
Biologic (%)	20
Glucocorticoid (%)	0

Supplementary Table 2. Clinical characteristics of patients who donated synovial biopsy samples from knee joint. CDAI; Clinical Disease Activity Index, CRP; C-Reactive Protein, ESR; Erythrocyte Sedimentation Rate. Other synthetic includes leflunomide and sulfasalazine. Biologic is etanercept.
